# A resorufin-based fluorescence probe for visualizing biogenic amines in cells and zebrafish†

**DOI:** 10.1039/d2ra06482k

**Published:** 2022-11-25

**Authors:** Sheng-Lin Pei, Jin Zhang, Wanyun Ge, Chao Liu, Ruilong Sheng, Lintao Zeng, Ling-Hui Pan

**Affiliations:** Department of Anesthesiology, Guangxi Medical University Cancer Hospital, Guangxi Clinical Research Center for Anesthesiology, Guangxi Engineering Research Center for Tissue & Organ Injury and Repair Medicine, Guangxi Health Commission Key Laboratory of Basic Science and Prevention of Perioperative Organ Disfunction Nanning 530021 China panlinghui@gxmu.edu.cn; School of Light Industry and Food Engineering, Guangxi University Nanning 530004 China zlt1981@126.com; CQM-Centro de Quimica da Madeira, Universidade da Madeira, Campus da Penteada Funchal 9000-390 Madeira Portugal

## Abstract

Biogenic amines (BAs) are a family of nitrogen-bearing natural organic molecules with at least one primary amine, which play an important role in living organisms. Elevated concentration of BAs may cause neuron disorder, Parkinson's disease and many other diseases. Therefore, it is essential to monitor BAs in living organisms. Herein, we reported a resorufin-based fluorescence probe for sensing of various BAs. Upon nucleophilic substitution reaction with BAs, the probe released resorufin, affording to strong fluorescence emission at 592 nm with rapid response (<8 min), good selectivity and a low detection limit (LOD = 0.47 μM). The probe has low cytotoxicity and good membrane permeability, and has been successfully used to visualize BAs in living cells and zebrafish with good performance.

## Abstract 2

A resorufin-based pre-fluorophore can achieve high contrast ratio imaging of biogenic amines in cells and zebrafish without washing steps.

## Graphical abstract

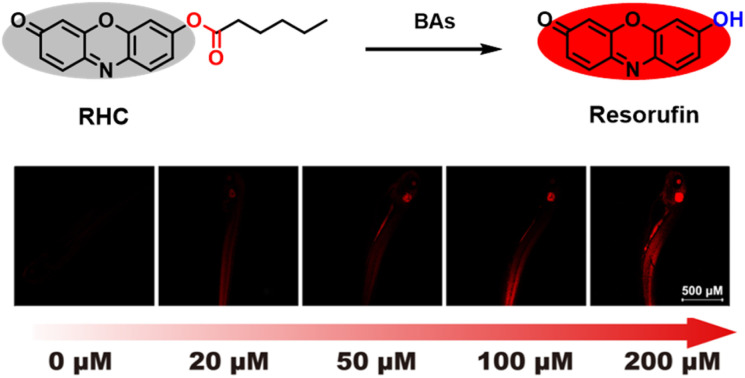

## 1 Introduction

Biogenic amines (BAs) are a family of nitrogen-bearing natural organic molecules with at least one primary amine group, which contain monoamines and polyamines. Monoamines mainly include tyramine, histamine, putrescine, cadaverine, phenylethylamine, tryptamine, *etc.* A certain amount of monoamines have diastolic and contractile effects on blood vessels and muscles,^1,2^ and have important regulatory effects on mental activities and cerebral cortex.^3^ Spermine and spermidine are the major components of polyamines, which can promote the synthesis of DNA, RNA and protein during the growth of organisms.^3^ BAs are indispensable components in living organisms and have important physiological functions in live cells.^4^ However, when the human body ingests excessive amounts of BAs, it can cause allergic reactions such as headache, nausea, palpitations,^5^ changes in blood pressure,^6^ as well as respiratory disturbances.^7^ Besides, BAs are also associated with various diseases, such as uterine cancer,^8^ lupus erythematosus,^9^ and liver disease.^10^ Therefore, the detection and identification of BAs are important issues for human health.

Fluorescence probe has evolved into an important tool to track biomolecules by imaging microscopy due to its advantages of simple operation, fast response, high selectivity, high sensitivity, and excellent spatial–temporal resolution.^11–17^

A couple of BAs-specific fluorescent probes have been developed on the basis of BAs-mediated nucleophilic reaction with aldehyde,^18–20^ ester^21^ and cyano group.^22,23^ However, these probes usually have strong background fluorescence interference, which cannot achieve high contrast ratio imaging of biogenic amines. Another approach is covalent coupling of amines with reactive fluorophores such as isothiocyanate,^24^*N*-hydroxysuccinimidyl (NHS) esters,^25^ and sulfonyl chloride groups.^26^ Nonetheless, these approaches are not ideal for the detection of amines due to the strong background fluorescence of fluorophores, which require washing steps to remove the excessive reactive fluorophore that did not react with amine. To eliminate the background fluorescence signals and avoid washing or distaining steps, an *in situ* activated fluorescent probe is preferable.^27,28^ In particular, it is highly desirable to create high brightness fluorophore with narrow absorption and emission bands within visible to near infrared (NIR) light region, since it could minimize parasitic signals from Rayleigh scattering (or auto-fluorescence) and reduce fluorescent resonance energy transfer (FRET)-based quenching under high concentration of local dyes.^21,29^ Moreover, pre-fluorophore approach, namely converting a non-fluorescent precursor to fluorophore through specific chemical reaction, might serve as a fast and selective sensing method to detect BAs *in situ*.

**Scheme 1:**
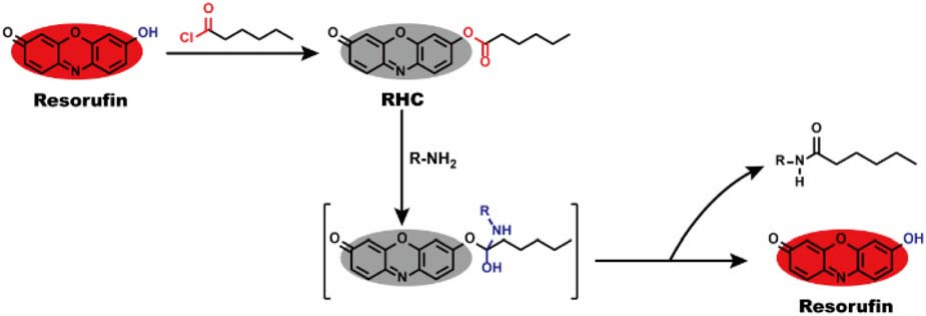
Design strategy and sensing mechanism of probe RHC to BAs.

Resorufin is a fluorophore with excellent performance including long wavelength fluorescence emission, high fluorescence quantum yield, good stability and strong anti-photobleaching ability, as well as low cytotoxicity and high biocompatibility,^30–33^ which enable it to be an efficient building block for creating fluorescent probes. By the virtue of these advantages, herein, we utilized resorufin as a starting material to prepare a fluorescent probe RHC for visualizing BAs. The caproyl group was attached to the hydroxyl group of resorufin to form caproyl ester group, which made the probe non-fluorescent and colourless. After reaction with BAs, the caproyl ester group of RHC was facilely removed through nucleophilic substitution reaction and thus released strong fluorescent resorufin dye (Scheme 1). The probe RHC exhibited an obvious fluorescence turn-on response towards BAs with rapid response, good selectivity, high sensitivity and low limit of detection (LOD). Besides, the probe has good biocompatibility and good sensing performance under physiological pH condition. Furthermore, the probe RHC has been successfully utilized to image BAs in living cells and zebrafish with good performance.

## 2 Materials and methods

### 2.1 Materials and general methods

Resorufin and hexanoyl chloride were bought from Sigma Aldrich and utilized without further purification. Cadaverine (Cad), putrescine, glutathione (GSH), cysteine (Cys), glucose, NaH_2_PO_4_ and Na_2_CO_3_ were purchased from Aladdin. Mouse fibroblast L929 cells were received from Perking Union Medical College, China. Zebrafish were supplied by Nanjing EzeRinka Biotechnology Co., Ltd China. ^1^H NMR and ^13^C NMR spectra were measured on a Bruker AVANCEIII 500 spectrometer at ambient temperature (Bruker, Billerica, MA). High-resolution mass spectra (HRMS) were determined on a HP-1100 LC-MS Spectrometer. UV-vis absorption spectra were performed on a SHIMADZU UV-800 spectrophotometer. Fluorescence spectra were measured on a Hitachi F-900 fluorescence spectrophotometer.

### 2.2 Preparation of probe RHC

Hexanoyl chloride (0.13 mL, 0.94 mmol) in 20 mL of anhydrous CH_2_Cl_2_ was added dropwise to resorufin (100.0 mg, 0.47 mmol) and K_2_CO_3_ (648.3 mg, 4.69 mmol) at ice-water bath under N_2_ atmosphere for 2 h. After quenching with saturated brine, the reaction solution was extracted with CH_2_Cl_2_. The crude product was purified by flash column chromatography (silica gel, petroleum ether/ethyl acetate, v/v = 3/1) to give RHC (65 mg, 53.4%) as a yellow powder. ^1^H NMR (500 MHz, CDCl_3_-d) *δ* ppm, 7.82 (d, *J* = 8.6 Hz, 1H), 7.46 (d, *J* = 9.8 Hz, 1H), 7.17 (s, 1H), 7.14 (d, *J* = 8.8 Hz, 1H), 6.89 (d, *J* = 9.8 Hz, 1H), 6.36 (s, 1H), 2.63 (t, *J* = 7.5 Hz, 2H), 2.37 (t, *J* = 7.6 Hz, 2H), 1.80 (m, 2H), 1.35 (m, 2H), 0.97 (t, *J* = 6.8 Hz, 3H). ^13^C NMR (125 MHz, CDCl_3_-d) *δ*/ppm 186.4, 171.4, 153.7, 149.4, 148.2, 144.4, 135.14, 134.8, 131.2, 125.0, 119.3, 109.7, 107.2, 34.3, 31.2, 29.7, 22.3, 13.9. HR-MS (ESI): calculated for [C_18_H_17_NO_4_ + H]^+^ 312.1236, found 312.1238.

### 2.3 Fluorescence imaging of BAs in living cells

L929 cells were seeded in confocal plates with the density of 2 × 10^4^ cells per well and incubated for 24 h. Then, RHC solution (10 μM) was added and incubated at 37 °C for 30 min. Afterward, the cells were washed twice with PBS, and various amounts of cadaverine were added and incubated for 15 min. Finally, fluorescence images were recorded on a Nikon A1 laser-scanning confocal microscope.

### 2.4 Fluorescence imaging of BAs in zebrafish

4 day old zebrafish were cultured in an E3 embryo medium containing RHC (10 μM) at 28 °C for 30 min. Then, the zebrafish were washed with E3 embryo medium for three times, and incubated with various amounts of cadaverine for 15 min. Finally, the fluorescence images were recorded on a Nikon A1 laser-scanning confocal microscope.

## 3 Results and discussion

### 3.1 Design and synthesis

Biogenic amines (BAs) contain at least one primary amine in their molecular structure, which has high nucleophilic activity to take aminolysis reaction with ester group. To develop a fluorescent probe for BAs, we used resorufin to prepare probe RHC, in which caproyl ester group acted as reactive recognition site for BAs. On the other hand, the caproyl ester group was attached to the oxygen atom of resorufin dye to mask the fluorescence of resorufin. In this case, the probe RHC was non-fluorescent and colourless. BAs would take nucleophilic substitution with the caproyl ester group to remove it. Consequently, the resorufin was released, affording strong red fluorescence. RHC was easily synthesized by routine method, and its structure was characterized by ^1^H NMR, ^13^C NMR and HR-MS (Fig. S1–S3, ESI†).

### 3.2 Spectral response of RHC towards cadaverine

**Fig. 1:**
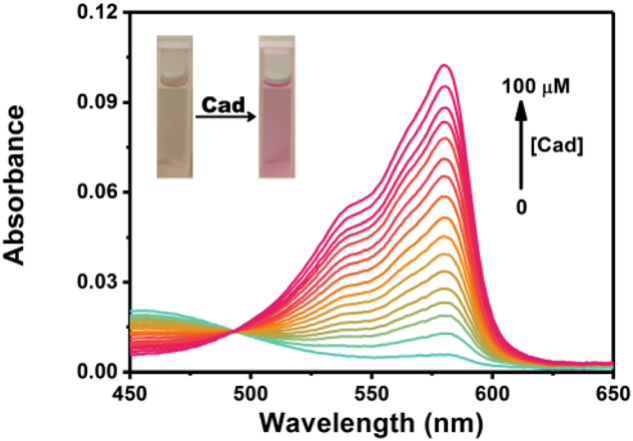
UV-vis absorption spectra of RHC (10 μM) in DMSO/PBS (v/v = 1 : 9, pH 7.4) upon treatment with cadaverine.

Cadaverine is one of typical BAs produced by enzyme-catalyzed decarboxylation of natural lysine in living organisms. Herein we chose cadaverine as a representative BAs to investigate the sensing performance of RHC. First, we examined the UV-vis absorption spectra and fluorescence responses of RHC towards cadaverine. RHC was pale yellow in PBS containing 10% DMSO. Upon the addition of cadaverine (0–100 μM), an obvious absorption peak appeared at 578 nm (Fig. 1). This absorption band gradually increased as increasing amount of cadaverine was added, affording an obvious color transition from colorless to pink. This apparent chromogenic effect facilitates the colorimetric determination of BAs.

**Fig. 2:**
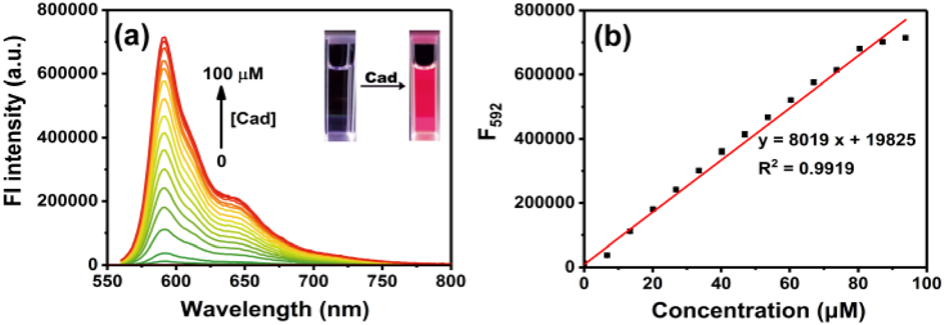
(a) Fluorescence spectra of RHC (10 μM) in DMSO/PBS (v/v = 1 : 9, pH 7.4, 10 mM) upon treatment with cadaverine at room temperature. (b) Calibration curve of fluorescence intensity at 592 nm *vs.* cadaverine (0–100 μM). The error bars indicate ±standard deviation. *λ*_ex_ = 550 nm, slits: 2/2 nm.

The probe RHC was intrinsic non-fluorescent (*Φ*_f_ < 0.002) in DMSO/PBS buffer (v/v = 1/9) due to the caproyl ester group, which masked the fluorescence of resorufin by protection of the phenol –OH. Upon the addition of cadaverine, a fluorescence band emerged at 592 nm. The fluorescence intensity became much stronger as the concentrations of cadaverine increased. Moreover, a good linear correlation was observed between the emission intensity (*F*_592_, *R*^2^ = 0.9919) and cadaverine concentrations (0–100 μM), and the detection limit (LOD) for cadaverine was 0.47 μM based on 3*σ*/*k*. By examining the absorption and emission spectra, we found that the absorption and fluorescence spectra of RHC after reaction with cadaverine were almost identical to resorufin,^30–33^ suggesting that resorufin was generated after the probe RHC reacted with cadaverine. The ^1^H NMR spectra of the product also supported our hypothesis. As shown in Fig. S4 (ESI†), there were three groups of aromatic proton signals at 7.23, 6.31 and 5.93 ppm, and a proton signal of –OH (Ha) was observed after RHC reacted with 3 equiv. of cadaverine, confirming the resorufin is the major product. The HR-MS spectra (Fig. S5, ESI†) also supported this hypothesis, where a predominant *m*/*z* = 213.0431 was attributed to resorufin [C_12_H_9_NO_3_]^+^. Based on this observation, we speculated that cadaverine reacted with the caproyl ester group of RHC to form amide and release resorufin. Therefore, obvious chromogenic and fluorogenic response of RHC towards cadaverine was observed (Fig. 2).

**Fig. 3:**
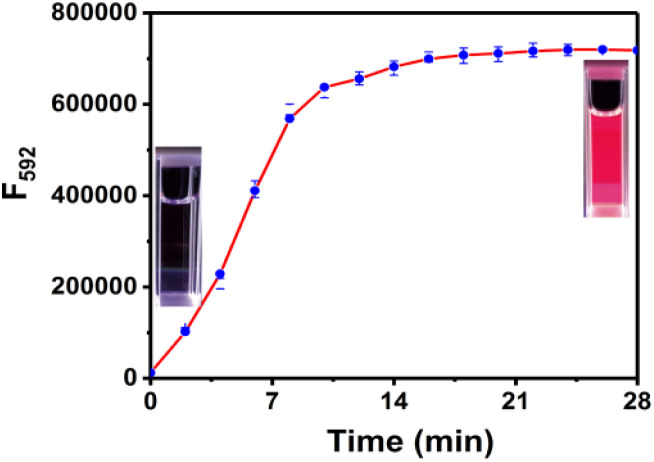
Time-dependent fluorescence intensity of RHC (10 μM) with cadaverine (100 μM). *λ*_ex_ = 550 nm, *λ*_em_ = 592 nm, slits: 2/2 nm. The error bars indicate ±standard deviation, *n* = 3.

**Fig. 4:**
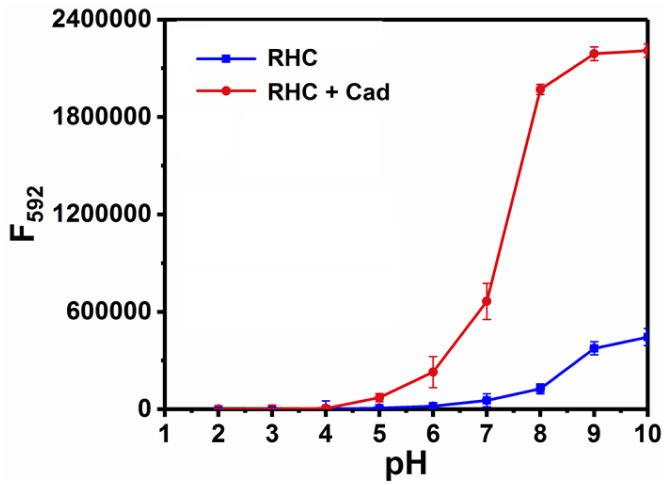
Fluorescence response of RHC (10 μM) towards cadaverine (100 μM) in aqueous solution (DMSO/PBS = 1/9, v/v) with different pH values. *λ*_ex_ = 550 nm, slits: 2/2 nm. The error bars represent ±SD, *n* = 3.

The time-dependent response of RHC to cadaverine was also investigated. RHC was almost non-fluorescent in DMSO/PBS buffer. Upon the addition of cadaverine at 25 °C, a significant fluorescence enhancement was achieved within 8 min (Fig. 3), suggesting that cadaverine could quickly activate the probe RHC to produce a strong red fluorescence. Besides, the pH-dependent fluorescence responses of RHC toward cadaverine were also investigated, as shown in Fig. 4. RHC has relatively weak fluorescence at pH 6.0–9.0, when the pH rose to 10.0, a basic hydrolysis of caproyl ester occurred and caused the release of resorufin, thus the pH range of 6.0–9.0 could be employed to conduct fluorescence measurements. Upon the addition of cadaverine (10 μM), the fluorescence intensity of RHC increased drastically at pH values ranging from 6.0–9.0, suggesting that RHC has good sensing performance towards cadaverine under physiological pH condition. Therefore, RHC might be used for fluorescence imaging of biogenic amines in cells and living organisms.

**Fig. 5:**
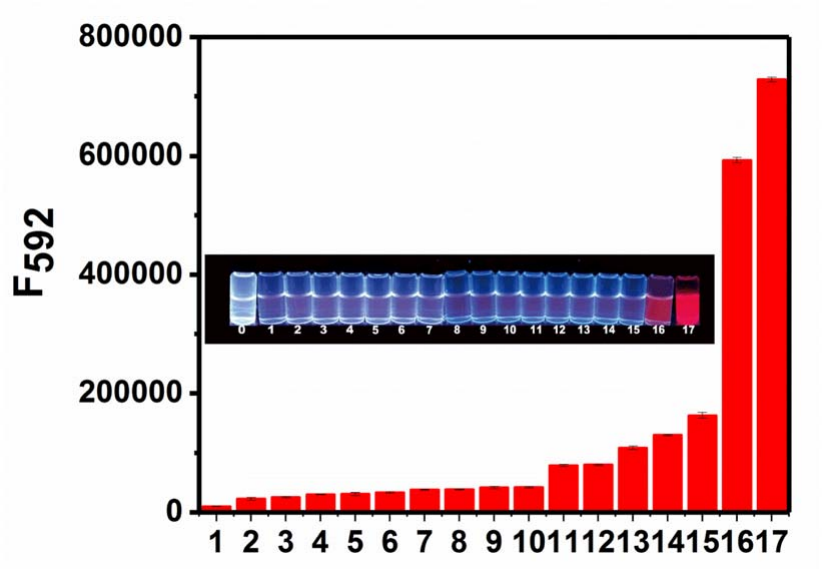
Fluorescence response of RHC (10 μM) toward various analytes (100 μM): (1) blank, (2) Cl^−^, (3) Na^+^, (4) GSH, (5) Cys, (6) HPO_4_^2−^, (7) HCO_3_^−^, (8) HS^−^, (9) glucose, (10) NO_3_^−^, (11) *N*,*N*-diisopropyl ethylamine, (12) triethylamine, (13) 4-methylpiperidine, (14) diethylamine, (15) ethylamine, (16) putrescine, (17) cadaverine, to RHC (10 μM) in DMSO/PBS (v/v = 1/9) at 25 °C. Inset: fluorescence photographs of RHC solution (10 μM) in the presence of various analytes. The photographs were recorded under 365 nm light.

To examine the selectivity of RHC for BAs, we measured the fluorescence response of RHC to various biological substances that generally exist in the living organisms, including biogenic amines (cadaverine and putrescine), organic amines (*N*,*N*-diisopropyl ethylamine, triethylamine, 4-methylpiperidine, diethylamine, and ethylamine), and other common biomolecules GSH, H_2_PO_4_^−^, HPO_4_^2−^, HCO_3_^−^, HS^−^, NO_3_^−^, glucose. As shown in Fig. 5, cadaverine and putrescine induced a large fluorescence enhancement. While the probe RHC showed a negligible response to Cl^−^, Na^+^, K^+^, Cys, GSH, HPO_4_^2−^, HCO_3_^−^, HS^−^, NO_3_^−^. Besides, ordinary organic amines such as *N*,*N*-diisopropyl ethylamine, triethylamine, 4-methylpiperidine, diethylamine, and ethylamine only induced much weaker fluorescence enhancement, which might be attributed to the stronger nucleophilicity of diamino-containing biogenic amines. These results indicated that RHC has good selectivity for cadaverine and putrescine over other amines and anions. Therefore, the probe RHC was favourable for imaging BAs in living cells and zebrafish.

### 3.3 Fluorescence imaging of BAs in living cells

**Fig. 6:**
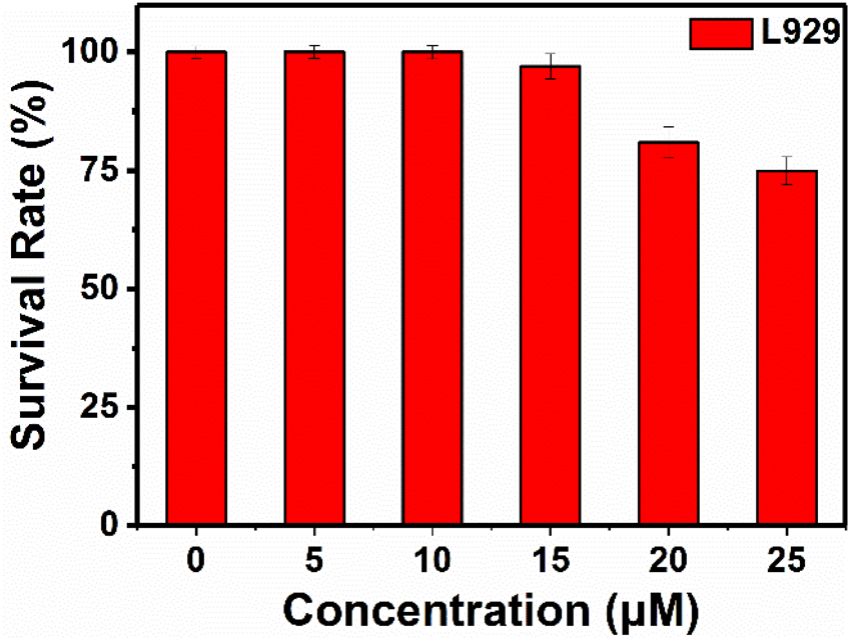
Cytotoxicity of RHC in L929 cells.

**Fig. 7:**
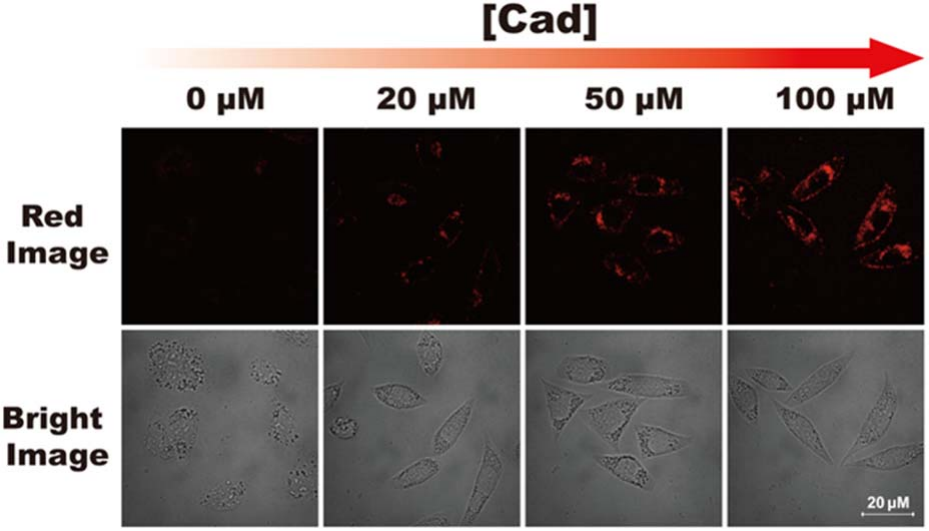
Confocal fluorescence images of RHC in L929 cells incubated with different concentrations of cadaverine. L929 cells were incubated with probe RHC (10 μM) at 37 °C for 16 min, and then further treated with various amounts of cadaverine at 37 °C for 16 min. *λ*_ex_ = 550 nm, *λ*_em_ = 560–650 nm. Scale bar: 20 μm.

Cytotoxicity of RHC were evaluated by CCK-8 assay before bio-imaging. As shown in Fig. 6, high cell viability (>92%) was observed after incubation with probe RHC (0–15 μM) for 24 h, indicating that RHC has good biocompatibility. Then, we studied the bio-imaging properties of RHC towards BAs in L929 cells. After incubation of RHC (10 μM) for 15 min, and the fluorescence images were recorded on a Nikon A1 laser-scanning confocal microscope. As shown in Fig. 7, negligible red fluorescence was observed from the red channel after incubation with RHC (10 μM). Then, the live cells were incubated with various amounts of cadaverine (20, 50, 100 μM) for 15 min and fluorescent images were recorded. When the RHC-containing L929 cells incubated with 20 μM of cadaverine, a weak red fluorescence image of cells was observed. As the concentration of cadaverine increased to 100 μM, much brighter red fluorescence in L929 cells was observed. The results demonstrated that probe RHC has a good bio-imaging capability towards cadaverine in living cells.

### 3.4 Fluorescence imaging of biogenic amine in zebrafish

**Fig. 8:**
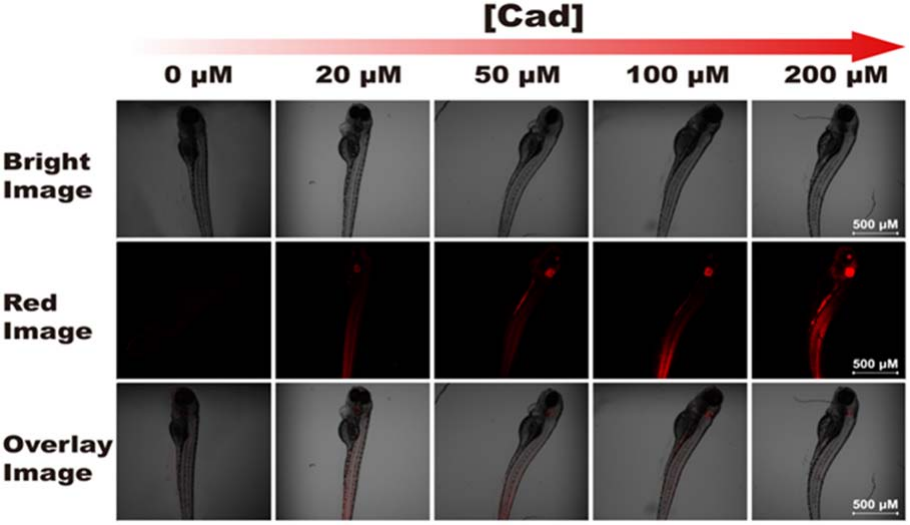
Confocal fluorescence images of probe RHC incubated with various amounts of cadaverine in zebrafish. *λ*_ex_ = 550 nm, *λ*_em_ = 560–650 nm. Scale bar: 500 μm.

Furthermore, we investigated the ability of probe RHC for imaging BAs *in vivo*. Herein, zebrafish was chosen as the research subject in this study, since zebrafish has 87% homologous genes with human body.^13,27^ As shown in Fig. 8, the zebrafish was non-fluorescent after incubation with RHC for 30 min, suggesting that the level of free biogenic amines was at a low level in zebrafish. Then, various amounts of cadaverine (20–200 μM) were treated with the zebrafish for 15 min, and the *in vivo* fluorescence images were obtained with laser confocal scanning microscope. As shown in Fig. 8, red fluorescence appeared in the gills and tail of the zebrafish when they were incubated with 20 μM of cadaverine for 15 min, and much brighter red fluorescence could be seen under the increased concentration of cadaverine (50–200 μM). These results demonstrated that probe RHC possessed good capability for fast and efficient *in vivo* imaging of BAs in zebrafish.

## 4 Conclusions

In summary, we successfully developed a fluorescent probe based on simply-structured resorufin dye for the sensing of biogenic amines. The probe taken a fast nucleophilic substitution reaction with biogenic amines, producing a chromogenic and fluorescence light-up response with excellent selectivity and sensitivity (LOD = 0.47 μM). Moreover, low cytotoxicity and good membrane permeability of probe RHC enable it serve as a good indicator for imaging biogenic amines both *in vitro* and *in vivo*. Therefore, the probe was successfully employed to *in situ* image the exogenous biogenic amines in zebrafish.

## Ethical statement

Fluorescence imaging in zebrafish were performed at the Department of Experimental Research from Guangxi Medical University Cancer Hospital. All zebrafish procedures were performed in accordance with the Guidelines for Care and Use of Laboratory Animals of Guangxi Medical University Cancer Hospital and approved by the Animal Ethics Committee of Guangxi Medical University Cancer Hospital.

## Author contributions

Sheng-Lin Pei: investigation, methodology, formal analysis, writing – original draft. Jin Zhang: investigation, formal analysis. Wanyun Ge: investigation, methodology. Chao Liu: investigation of sensing mechanism. Ruilong Sheng: conception, writing – review & editing. Lintao Zeng: methodology, formal analysis, writing – original draft. Ling-Hui Pan: conception, supervision, writing – review & editing.

## Conflicts of interest

There are no conflicts to declare.

## Supplementary Material

RA-012-D2RA06482K-s001
