# Supplementary material for: A resorufin-based fluorescence probe for visualizing biogenic amines in cells and zebrafish
Source: RSC Adv. 2022 Nov 25;12(52):33870–5. doi: 10.1039/d2ra06482k (PMC9693732; doi:10.1039/d2ra06482k)
Supplement: RA-012-D2RA06482K-s001 [file RA-012-D2RA06482K-s001.pdf]

Electronic Supplementary Material (ESI) for RSC Advances.
This journal is © The Royal Society of Chemistry 2022

Electronic Supplementary Information

A resorufin-based fluorescence probe for visualizing biogenic amines in cells and zebrafish

Sheng-Lin Pei,[1] Jin Zhang,[2] Wanyun Ge,[1] Chao Liu,[1] Ruilong Sheng,[3] Lintao Zeng,[2,*] Ling-Hui Pan[1,*]

[1] *Department of Anesthesiology, Guangxi Medical University Cancer Hospital; Guangxi Clinical Research Center for Anesthesiology; Guangxi Engineering Research Center for Tissue & Organ Injury and Repair Medicine; Guangxi Health Commission Key Laboratory of Basic Science and Prevention of Perioperative Organ Disfunction, Nanning 530021, China.*

[2] *School of Light Industry and Food Engineering, Guangxi University, Nanning 530004, China.*

[3] *CQM-Centro de Quimica da Madeira, Universidade da Madeira, Campus da Penteada, Funchal 9000-390, Madeira, Portugal.*

**Corresponding authors:*

Email: panlinghui@gxmu.edu.cn (L.-H. Pan); zlt1981@126.com (L. Zeng).

Table of Contents

Experimental details

Fig. S1. ^{1}H NMR spectra of **RHC** in $CDCl_3$-*d* (500 MHz).

Fig. S2. ^{13}C NMR spectra of **RHC** in $CDCl_3$-*d* (125 MHz).

Fig. S3. HR-MS spectra of **RHC**.

Fig. S4. ^{1}H NMR spectra of **RHC** after reaction with cadaverine in D_2O (500 MHz).

Fig. S5. HR-MS spectra of **RHC** after reaction with cadaverine.

1. Experimental details

1.1 Spectral analysis

First, **RHC** (5.0 mM) stock solution was prepared by dissolving **RHC** solid in pure DMSO (99.9%). Before measurements, the **RHC** stock solution was diluted with DMSO/PBS buffer solution (pH= 7.4, v/v = 3/7) at room temperature to prepare working solution (10 μM). The UV-vis absorption spectra were measured on a spectrophotometer (SHIMADZU UV-800), and fluorescence spectra were recorded on fluorescence spectrometer (Hitachi F-900). λ_{ex} = 550 nm, λ_{em} = 592 nm, slits: 2/2 nm.

1.2 Cell Culture and Cytotoxicity assay

L929 cells were cultured in DMEM with 10% FBS and penicillin (100 units/mL)-streptomycin (100 μg/mL) liquid at 37 ^{o}C in a humidified incubator containing 5% CO_2 in air. For cytotoxicity analysis, L929 cells were first seeded in a 96-well plate (Corning, USA) with the density of 5.0× 10^3 cells/per well.

Cytotoxicity of probe **RHC** was determined using CCK-8 assay in L929 cells. After incubation for 24 h, the 96-well plate was washed with 100 μL/well PBS and the L929 cells were incubated with different amount of **RHC** (0, 5, 10, 15, 20, and 25 μM) for 24 h. Afterward, the cells were washed with serum-free DMEM once, and then serum-free DMEM containing 10% CCK-8 (100 μL) was added to each well and incubated for 1 h. The absorbance (λ_{abs} =450 nm) was measured on a microplate reader. Cell viability (%) was calculated according to literature.

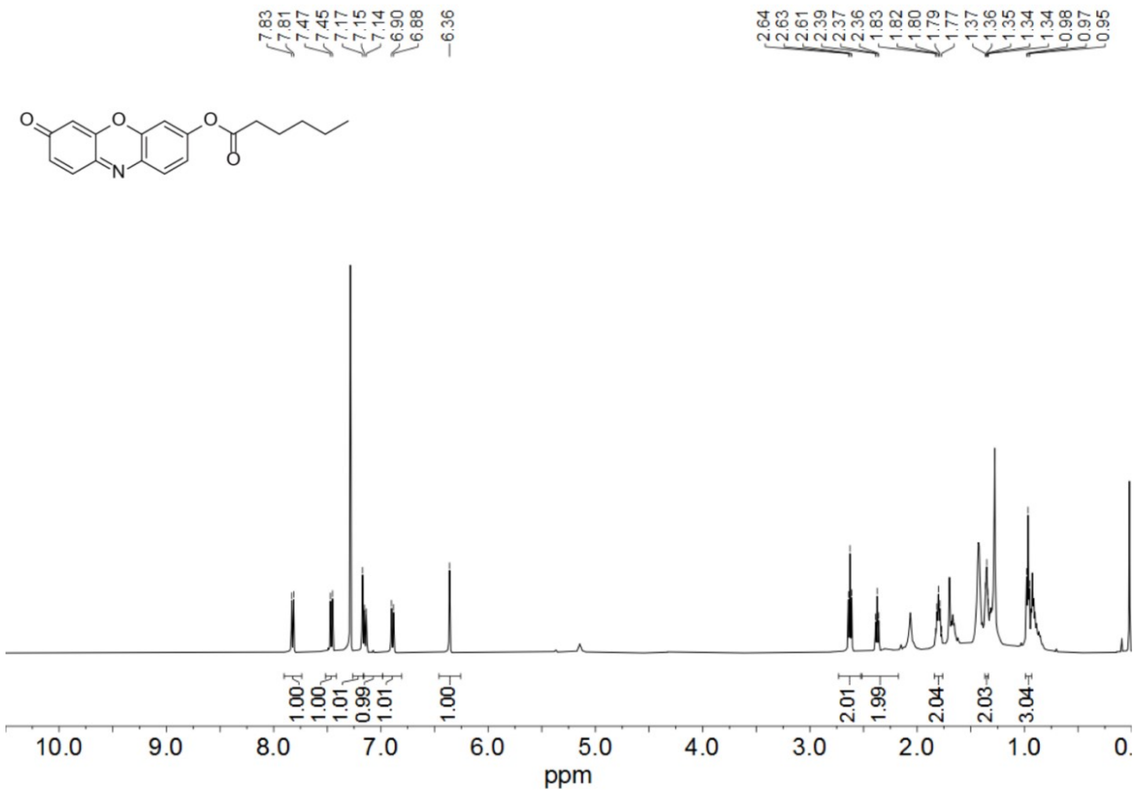

Fig. S1. ^{1}H NMR spectra of **RHC** in $CDCl_3$-*d* (500 MHz).

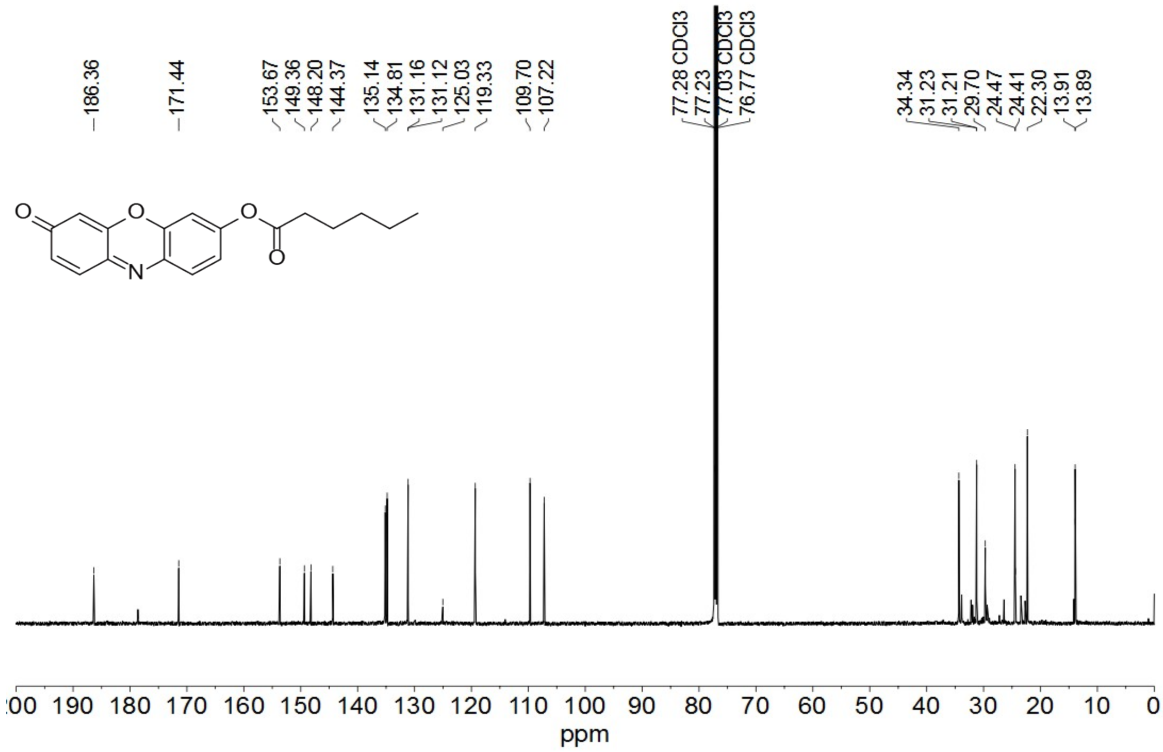

Fig. S2. ^{13}C NMR spectra of **RHC** in $CDCl_3$-*d* (125 MHz).

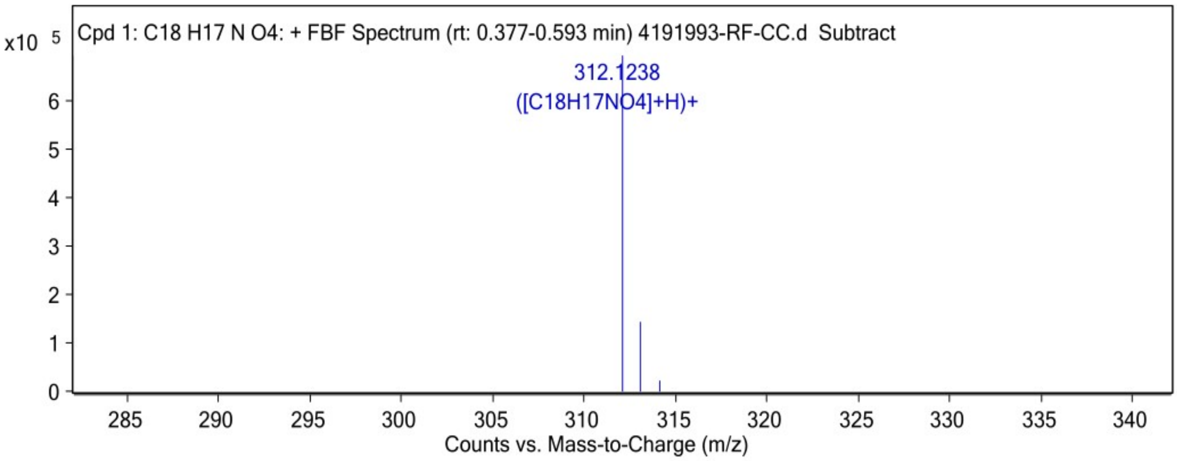

Fig. S3. HR-MS spectra of **RHC**.

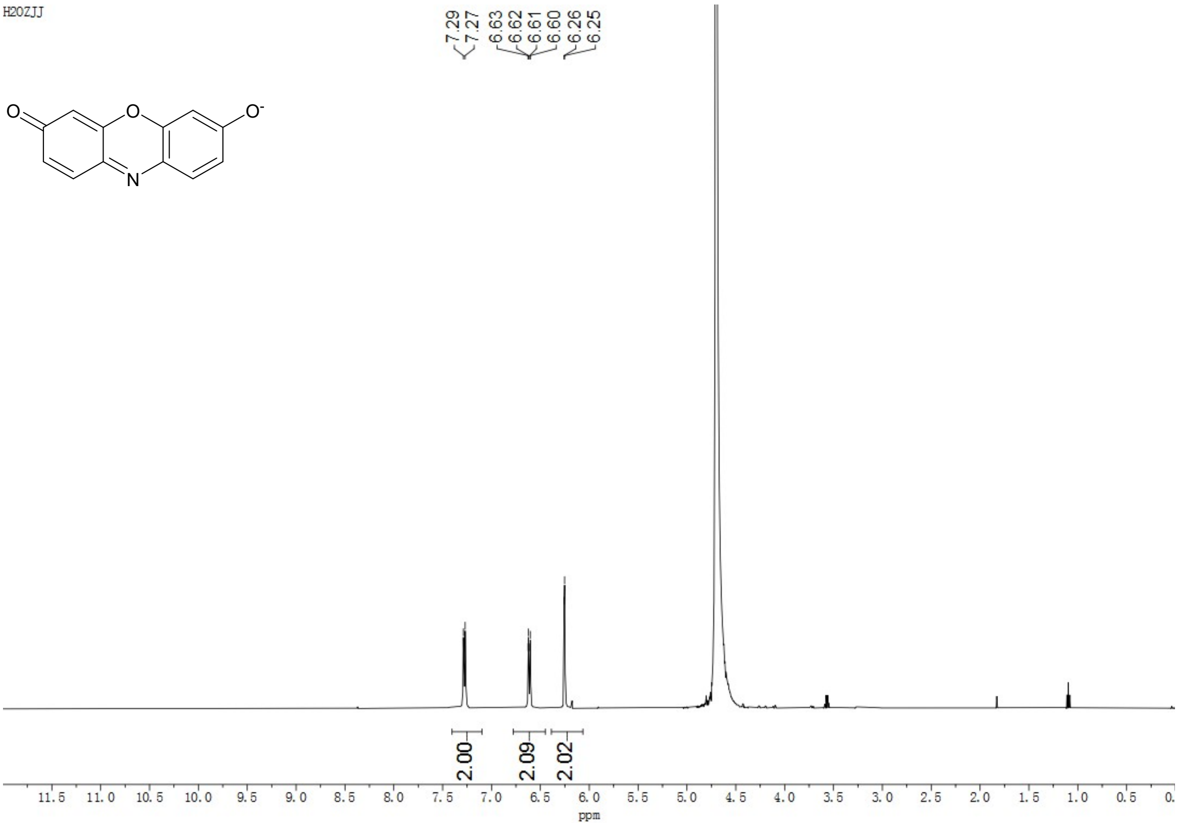

Fig. S4. ^{1}H NMR spectra of **RHC** after reaction with cadaverine in D_2O (500 MHz).

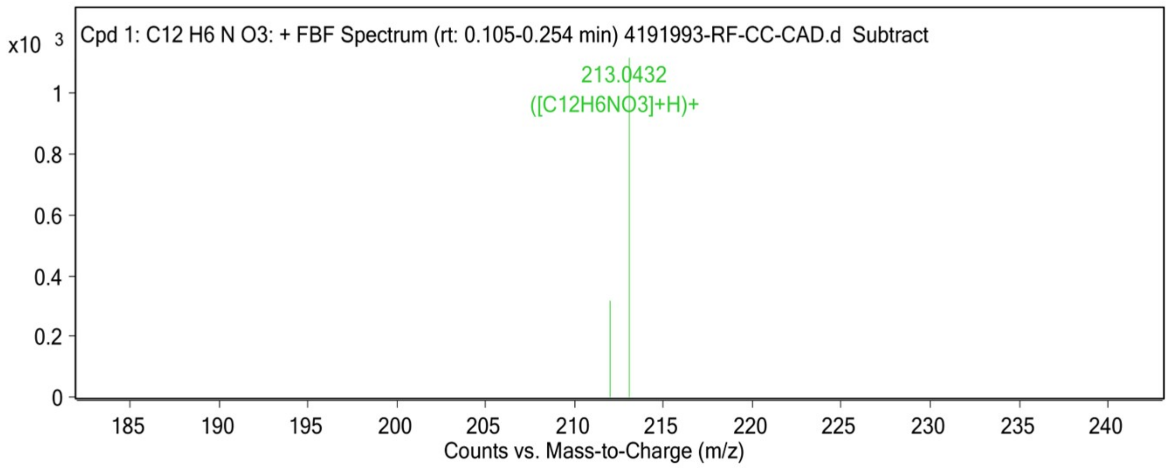

Fig. S5. HR-MS spectra of **RHC** after reaction with cadaverine.
